# A case of delayed perforation after cold snare polypectomy treated conservatively by endoscopic clip closure

**DOI:** 10.1002/deo2.302

**Published:** 2023-10-20

**Authors:** Toshifumi Iida, Yohei Minato, Susumu Banjoya, Tomoya Kimura, Koichi Furuta, Shinya Nagae, Yohei Ito, Hiroshi Yamazaki, Nao Takeuchi, Shunya Takayanagi, Yuki Kano, Takashi Sakuno, Kohei Ono, Ken Ohata

**Affiliations:** ^1^ Department of Gastrointestinal Endoscopy NTT Medical Center Tokyo Tokyo Japan

**Keywords:** cold snare polypectomy, delayed perforation, endoscopic hemo‐clip closure, endoscopic clip closure, sigmoid colon

## Abstract

We present the case of a 45‐year‐old man who underwent a screening total colonoscopy and developed delayed perforation after a cold snare polypectomy in the descending colon and sigmoid colon. The patient developed abdominal pain and was referred to our department for further evaluation and treatment. On clinical examination, we noted lower abdominal tenderness, mild rebound pain, and elevated levels of inflammatory markers. Abdominal contrast‐enhanced computed tomography confirmed the presence of free air in the abdomen. Since there were no signs of peritoneal inflammation and the vital signs were stable, we planned to perform endoscopic clip closure of the perforated area. The patient's symptoms improved with conservative management thereafter, including fasting, fluid replacement, and antibiotic administration. The patient was discharged on the 6th hospital day. In this case report, we discuss the usefulness of endoscopic clip closure in managing delayed perforation.

## INTRODUCTION

Cold snare polypectomy (CSP) was first reported in 1992 as a safe and effective treatment for small polyps.^1^ As compared to hot snare polypectomy, CSP offers the advantage of resection of more superficial mucosa while carrying a lower risk of perforation.^2^, ^3^ There have been no reported cases of delayed perforation after CSP. This case presented herein is the first of delayed perforation occurring after CSP that was successfully managed by endoscopic hemo‐clip closure.

## CASE REPORT

The patient was a 45‐year‐old man who presented to a neighborhood hospital with the chief complaint of lower abdominal pain, and a colonoscopy was planned. As for the history of present illness, colonoscopy was performed, followed by CSP for a 4‐mm 0‐IIa lesion in the descending colon and a 6‐mm 0‐IIa lesion in the sigmoid colon. The snare used in this procedure was 10 mm in size (high‐frequency snare, Micro‐Tech Co.).

Following these procedures, the patient returned home with no subjective symptoms such as abdominal pain, not to mention findings suggestive of peritonitis. However, about 3 h after the procedure, the patient developed abdominal pain after consuming his first meal. Therefore, he returned to his prior hospital and was referred to our department on the same day. At our department, a physical examination revealed abdominal tenderness and mild rebound pain. By the time of the patient's visit to our department, 6 h had already passed since the CSP procedure. The patient's past medical history included dyslipidemia, hyperuricemia, and allergic rhinitis, and the patient was under regular treatment with oral levocetirizine. He was not in the habit of smoking or drinking.

During the physical examination, we noted tenderness and mild rebound pain in the lower abdomen. There was no fever or hypotension. The results of the blood tests conducted at this time were as follows: white blood cells, 12,000/μL, red blood cells 533 × 104/μL, hemoglobin 16.6 g/dL, platelet count 27.2 × 104/μL, blood urea nitrogen 16.2 mg/dL, serum creatinine 0.94 mg/dL, serum aspartate transaminase 29 IU/L, serum alanine transaminase 39 IU/L, serum Na 141 mEq/L, serum K 3.5 mEq/L, serum Cl 103 mEq/L, and serum C‐reactive protein <0.30 mg/dL.

**FIGURE 1:**
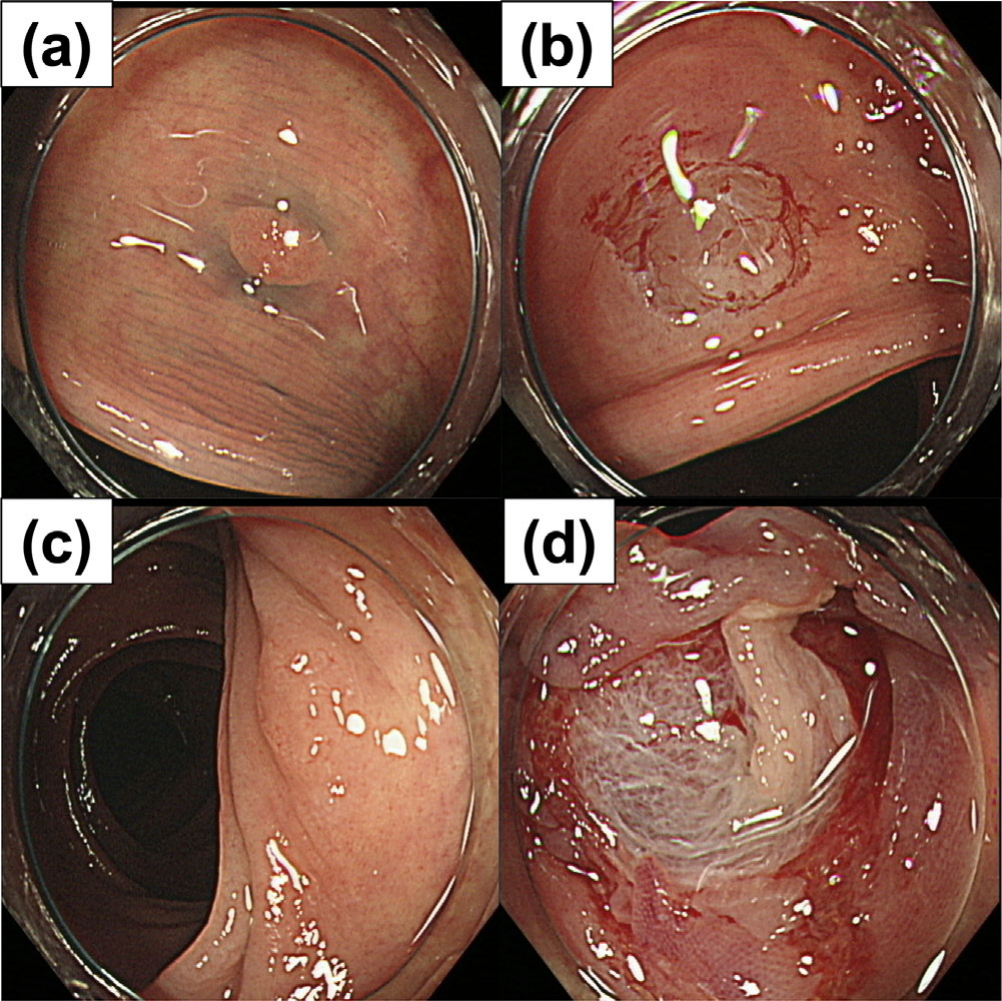
The endoscopic findings at the previous clinic. (a) 4‐mm 0‐IIa lesion in the descending colon. (b) The descending colon polyp after cold snare polypectomy. (c) 6‐mm Is lesion of the sigmoid colon. (d) The ulcer base in the sigmoid colon.

Figure 1 shows the endoscopic findings recorded at the referring institution. CSP was performed on a 4‐mm 0‐IIa lesion in the descending colon (Figure 1a,b) and 6‐mm 0‐IIa lesion in the sigmoid colon (Figure 1c,d). The lesion was a 6 mm flat elevated lesion with no evidence of submucosal invasion. There was no fibrosis of the lesion. It is unclear whether the muscle layer was gripped during snaring. But it wasn't turn off forcibly without feeling any resistance. The ulcer base after treatment was not large. The CSP was performed in a short period of time. CO_2_ was used for air delivery. Based on these endoscopic images and evaluation of the ulcer base, no clear sign of intraoperative perforation could be confirmed.

**FIGURE 2:**
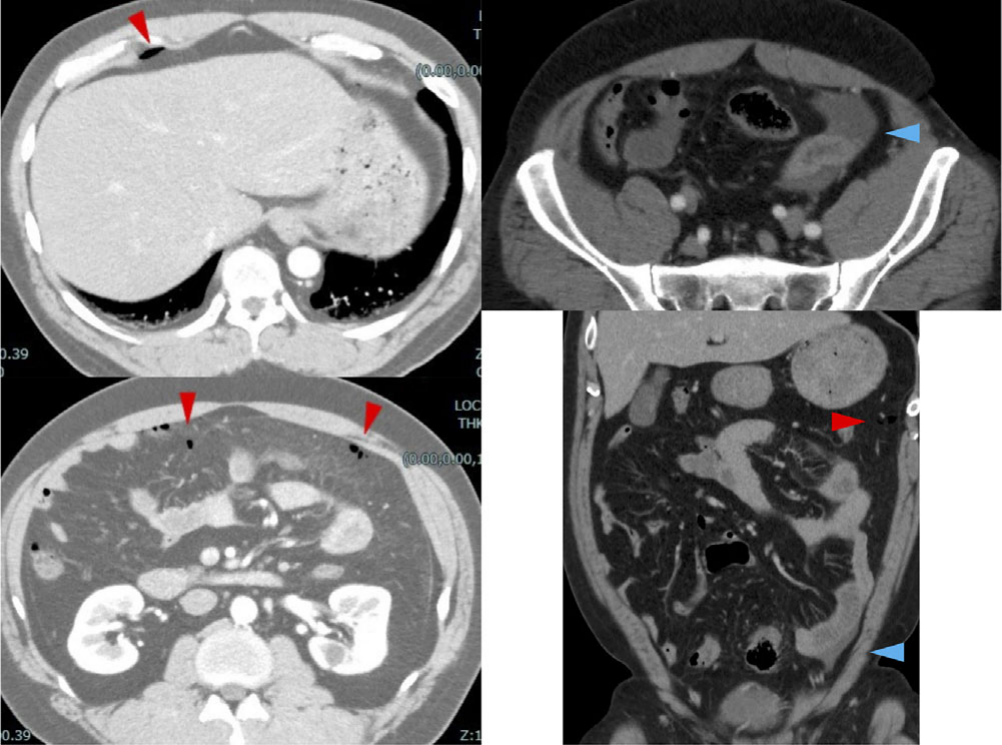
Contrast‐enhanced abdominal computed tomography at our hospital. Red arrows indicate free air. Blue arrows indicate ascites.

We performed contrast‐enhanced abdominal computed tomography at our hospital (Figure 2). Although it was difficult to identify the perforated site on the computed tomography images, free air in the abdomen was observed in multiple areas, including the upper abdomen and abdominal cavity. There was no extravasation of the contrast agent or evidence of intestinal ischemia/intestinal necrosis. The patient's overall condition remained stable after the CSP, and he developed abdominal pain only shortly after his first meal after the procedure. Based on these findings, we suspected delayed perforation after the CSP, and in consultation with a surgeon, decided to perform endoscopic clip closure of the perforated area of the bowel.

**FIGURE 3:**
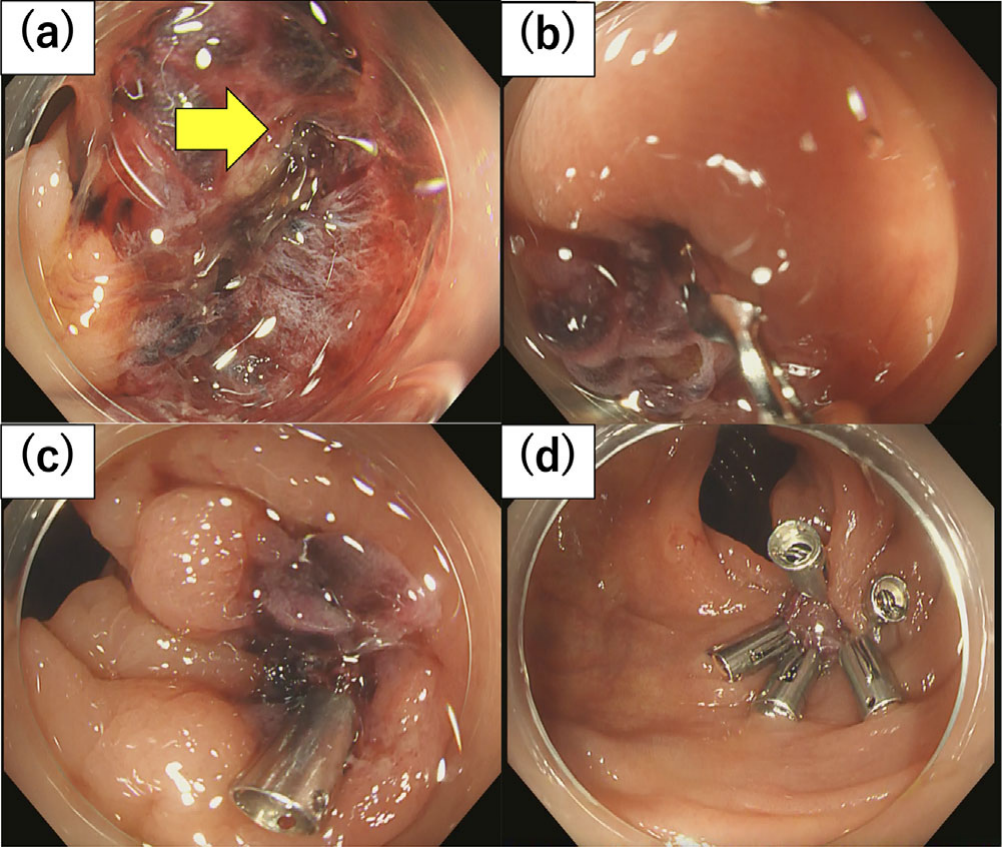
The endoscopic findings at our department. (a) The ulcer base in the sigmoid colon. Perforation site. (b–d) Suture was performed using six hemoclips.

The endoscopic examination confirmed perforation at the site of the CSP in the sigmoid colon. We performed endoscopic clip closure of this perforated area using six hemo‐clips (Figure 3 and Video S1).

Following admission to our hospital, conservative management included fasting, fluid replacement, and antibiotic administration. Initially, there was a temporary increase in the inflammatory response marker levels and fever, but gradual improvement of the signs and laboratory values was observed over time. On postoperative day 5 (POD 5), the patient was started on oral intake, and after confirming that he did not develop any abdominal pain, he was discharged on POD 6. At the follow‐up visit one month after discharge from our hospital, the patient reported no clear recurrence of the abdominal pain and laboratory examination revealed no abnormalities in his inflammatory response marker levels.

**FIGURE 4:**
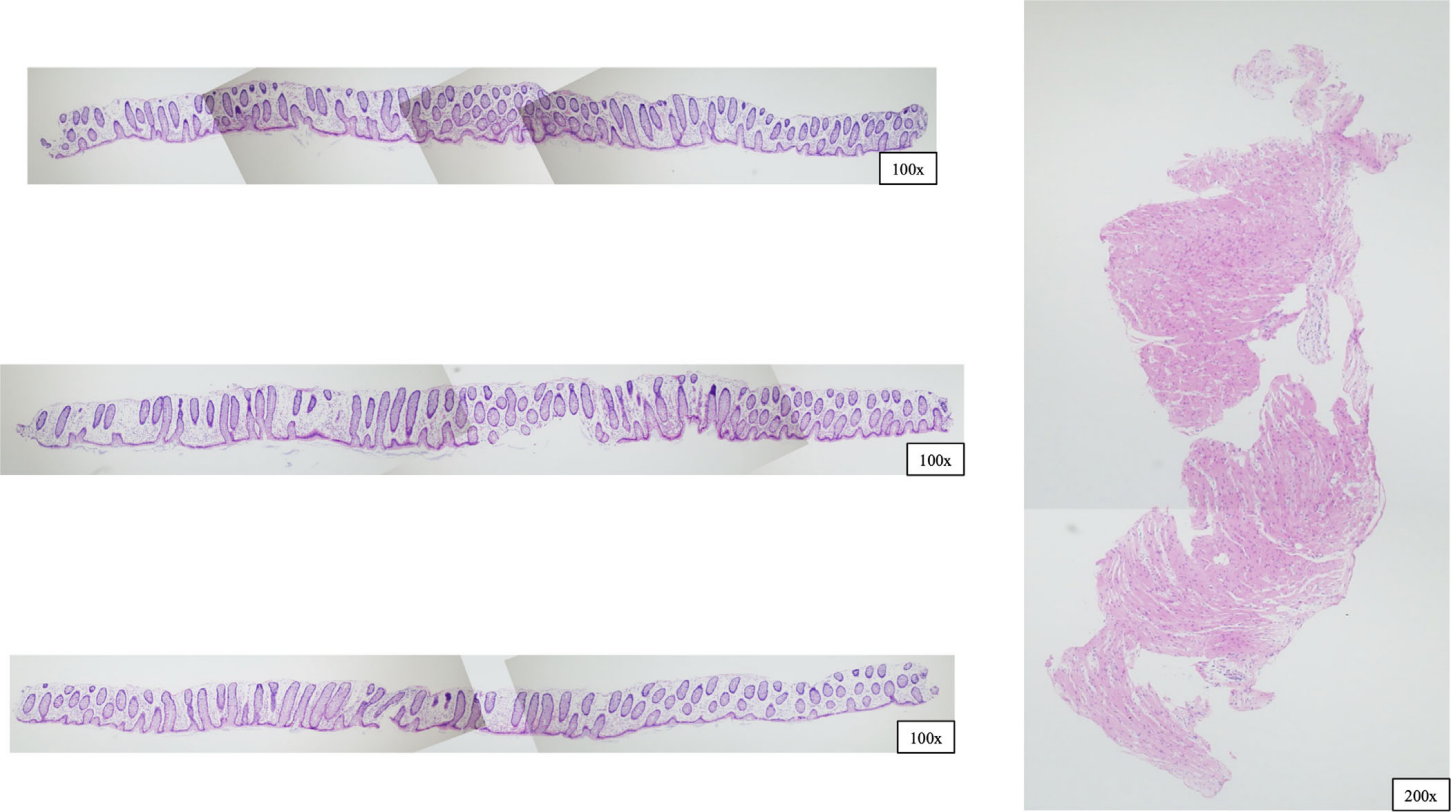
The sigmoid colon specimen. The specimen contained muscularis propria, but not the serosa.

We present the resection specimen and pathological results of the sigmoid colon polyp resected by CSP in this case. The specimen was fragmented upon collection. Four tissue sections were obtained from the sigmoid colon specimen, with one section showing adhesion of the intrinsic muscularis propria, while none of the submitted specimens contained serosa (Figure 4). Histopathology revealed mildly inflamed mucosa with no neoplastic changes. It is unclear why part of the muscle layer was removed.

## DISCUSSION

To date, there has been only one reported case of delayed perforation after CSP, by Iwano et al. in 2022.^4^ However, in that particular case, on the day after the procedure, the patient had developed postoperative hemorrhage, which was controlled by the use of additional clips at the suspected sites of bleeding, but not at the site of the delayed perforation. Moreover, the interval between the CSP and delayed perforation was as long as 8 days. This extended interval raises the possibility that the subsequent endoscopic procedures might also have contributed to the occurrence of the delayed perforation.

Therefore, it is difficult to determine if delayed perforation occurs solely as a complication of CSP, or if any additional procedures conducted thereafter might also contribute to the development of delayed perforation. In our present case, no perforation was observed immediately after the CSP and the diagnosis of delayed perforation was confirmed only after the subsequent onset of abdominal pain, computed tomography evidence of the presence of free air in the abdomen, and endoscopic confirmation of the perforation site. Furthermore, unlike the unfortunate outcome of death in the case reported by Iwano et al., we could successfully manage our patient through endoscopic treatment.

In this case, since partial resection of the muscularis propria was performed during CSP, the possibility that microperforation occurred during treatment and was manifested by the first meal is also considered. However, no perforation was observed during the postoperative confirmatory endoscopic examination. In addition, considering the patient's clinical course of sudden abdominal pain and the subsequent identification of a clear perforation site by colonoscopy in our hospital, we categorized the case as one of delayed perforation after CSP. One possible reason why peritonitis did not develop in this case is that the perforation was very small. We think Another factor was the rapidity of the process from discovery to treatment.

de'Angelis et al. have reported that more invasive treatment, such as surgery, is required in patients with a more than 24‐hour delay in the diagnosis of medically induced perforation.^5^ Panteris et al. reported that emergency surgery is often recommended when there is a risk of major perforation and signs of peritonitis or abdominal symptoms.^6^ According to a previous report, surgical treatment is typically considered the primary choice of treatment for delayed perforation. However, based on the idea that CSP is associated with less thermal degeneration of the mucosa and our own experience with this case, we propose that endoscopic clip closure can be considered as a potential treatment option for cases of delayed perforation, if the patient's blood pressure, consciousness level, and other vital signs are stable and the effects of the pre‐treatment bowel cleansing are still expected to be maintained. In this case, the patient developed early after the procedure was performed, and it was possible to perform the procedure without applying any pretreatment.

This case is significant as it represents the first successful instance of endoscopic clip closure for delayed perforation after CSP, namely, of successful conservative improvement.

## CONFLICT OF INTEREST STATEMENT

None.

## ETHICS STATEMENT

All procedures followed have been performed in accordance with the ethical standards laid down Declaration of Helsinki and its later amendments.

## Supporting information

Video S1 The endoscopic findings at our department. Suture was performed using six hemoclips.
